# Effectiveness of Subacromial Methylprednisolone Acetate Injection With Lignocaine in Rotator Cuff Tendonitis

**DOI:** 10.7759/cureus.84383

**Published:** 2025-05-19

**Authors:** Asif Afridi, Midrarullah Khan, Zeeshan Q Qamar

**Affiliations:** 1 Trauma and Orthopaedics, Hayatabad Medical Complex Peshawar, Peshawar, PAK; 2 Trauma and Orthopaedics, University Hospitals Birmingham NHS Foundation Trust, Birmingham, GBR; 3 Orthopaedics and Spine, Hayatabad Medical Complex Peshawar, Peshawar, PAK

**Keywords:** 2% lignocaine, clinical effectiveness, methylprednisolone, rotator cuff tendonitis, subacromial bursa steroid injection

## Abstract

Background

The rotator cuff, composed of subscapularis, infraspinatus, teres minor, and supraspinatus muscles, provides stability and a wide range of motion to the glenohumeral joint. Accurate diagnosis and appropriate treatment are crucial for effective management. This study aimed to assess short-term clinical outcomes, including reduction in pain and improvement in shoulder function, following subacromial injection of methylprednisolone acetate combined with lignocaine in patients with rotator cuff tendonitis.

Methodology

This descriptive study was conducted at the Department of Orthopedic and Spine, Hayatabad Medical Complex, Peshawar, over six months (May to November 2023). A total of 87 patients were treated with 80 mg of methylprednisolone acetate diluted in 4 mL of 2% lignocaine, injected into the subacromial space. Follow-ups at three weeks assessed effectiveness.

Results

The mean age was 46 years (SD = ±12.10), with 46% male and 54% female patients. Pain was reported in the right shoulder in 56% of cases. The mean duration of complaints was three weeks (SD = ±1.27). The intervention was effective in 81 (93%) patients.

Conclusions

Subacromial methylprednisolone acetate injection with lignocaine demonstrated a high rate of short-term symptom improvement (93%) in managing rotator cuff tendonitis.

## Introduction

The rotator cuff is a group of muscles made up of the subscapularis, infraspinatus, teres minor, and supraspinatus muscles, which allow a wide range of motion and stability to the glenohumeral joint [1]. Inflammation of the tendons of these muscles is referred to as rotator cuff tendonitis [2]. Often present along with shoulder impingement, it has many common alternative terms such as tendinosis, rotator cuff fraying, partial thickness tears, and tendonitis. It is one of the frequent problems among patients presenting with the complaint of shoulder pain, which may present acutely after an injury or may be due to repetitive overuse activity of the joint [3,4]. The incidence of rotator cuff tendonitis increases with an aging population, accounting for approximately 44%-66% of all the shoulder-related pain, and is an important cause of disability of the shoulder joint [5,6]. Non-surgical treatment guidelines are mainly recommended initially with exercise physiotherapy, analgesics, or subacromial steroid injection. Intra-articular injections can be easily performed in outpatient departments. Steroids are widely used in intra-articular injections for the treatment of shoulder pain because of their strong anti-inflammatory effects. Despite these benefits, trials are still needed to uniformly address the anatomical site, frequency, dose, and type of steroids [7-9]. Hall et al. reported response rates for the primary outcome of 94% at six weeks, 88% at six months, and 80% at 12 months [10]. Currently, limited studies have addressed this pathology regarding subacromial methylprednisolone injection with local anesthetic in the Pakistani population. This study aims to review the evidence of the effectiveness and safety of subacromial steroid injections with local anesthetic for the treatment of shoulder pain or rotator cuff tendonitis. The study findings will be helpful in generating local evidence regarding the efficacy of subacromial steroid injections with local anesthetic for general practitioners, family physicians, rheumatologists, and orthopedic surgeons. Taking these issues into account, along with correct etiological diagnosis and choice of treatment, is essential for good outcomes.

## Materials and methods

This study was conducted at the Department of Orthopedic and Spine, Hayatabad Medical Complex, Peshawar, over six months, from May 14, 2023, to November 14, 2023. This descriptive study was designed to assess the effectiveness of subacromial methylprednisolone acetate injections with lignocaine for the treatment of rotator cuff tendonitis. Effectiveness was defined as a composite outcome of pain reduction and functional improvement, assessed through the Visual Analog Scale (VAS) for pain at baseline and the three-week follow-up. A reduction of ≥2 points on the VAS was considered clinically meaningful. Functional improvement was assessed qualitatively during clinical examination based on the patient’s ability to perform daily activities such as combing hair, reaching overhead, and dressing independently. Methylprednisolone acetate was selected due to its intermediate duration of action, established anti-inflammatory efficacy, and wide clinical use in musculoskeletal conditions. Its pharmacokinetics allow for sustained local effect while minimizing systemic absorption. Previous studies have demonstrated its effectiveness in managing subacromial pain syndromes, making it a commonly preferred agent among clinicians. The sample size of 87 patients was determined using the World Health Organization sample size calculator, based on a 94% expected effectiveness rate, a 95% confidence interval, and 5% precision. A non-probability consecutive sampling method was employed to recruit participants, ensuring that all eligible patients who met the study criteria during the study period were included.

Inclusion criteria

Both male and female patients aged 20-60 years who were diagnosed with rotator cuff tendonitis were considered for inclusion in the study. Patients with a Constant-Murley Score >30, indicating moderate to severe symptoms, were included.

Exclusion criteria

Patients with complex shoulder trauma, recent shoulder injections, or prior shoulder surgery were excluded. Additionally, patients with conditions such as rheumatoid arthritis, use of anticoagulants, immunocompromised status, pregnancy, diabetes, or hypertension were also excluded.

Data collection procedure

After receiving ethical approval, patients who met the inclusion criteria were recruited from the outpatient department and the Department of Orthopedic and Spine. Written informed consent was obtained from all participants before their inclusion in the study. Detailed medical histories, clinical examinations, and radiological assessments were performed to confirm the diagnosis and determine the severity of rotator cuff tendonitis. Each participant was administered a subacromial injection of 80 mg methylprednisolone acetate (2 mL) diluted with 4 mL of 2% lignocaine hydrochloride under sterile conditions. To reduce confounding, patients were instructed to avoid the use of additional treatments such as oral analgesics, physical therapy, or home-based shoulder exercises during the three-week follow-up period unless absolutely necessary. Compliance with this instruction was confirmed at follow-up visits through patient self-reporting. Follow-up assessments were conducted at three weeks to evaluate the effectiveness of the treatment in terms of symptom relief and functional improvement. Demographic and clinical data, including patient age, gender, weight, and the duration of complaints, were systematically recorded on a structured proforma.

Injection procedure

Each participant received a subacromial injection of 80 mg methylprednisolone acetate (2 mL) diluted in 4 mL of 2% lignocaine hydrochloride using a landmark-guided posterior approach, under aseptic conditions.

Data analysis

Data were analyzed using SPSS version 23 (IBM Corp., Armonk, NY, USA). Descriptive statistics were calculated, including mean and standard deviation (SD), for numerical variables such as age, weight, and duration of complaints. Categorical variables, including gender, side of the shoulder, and treatment effectiveness, were summarized using frequencies and percentages. Effectiveness was stratified by demographic factors such as age, gender, weight, and duration of complaints. The chi-square test was applied post-stratification to assess the association between treatment effectiveness and demographic factors, with a significance threshold set at p-values <0.05. The results of the statistical analysis are presented in tables and charts for clear visualization and interpretation of the findings.

## Results

In this study, 43 (49%) patients were in the age group of 20-40 years, and 44 (51%) patients were in the age group of 41-60 years. The mean age was 46 years (SD = ±12.10). Overall, 40 (46%) patients were male, and 47 (54%) patients were female. Further, 36 (41%) patients weighed ≤80 kg, and 51 (59%) patients weighed >80 kg. The mean weight was 80 kg (SD = ±10.08). In total, 38 (44%) patients had pain on the left side of the shoulder, and 49 (56%) patients had pain on the right side of the shoulder (Table 1).

**Table 1:** Demographic characteristics of participants (n = 87).

Variable	Categories	Frequency	Percentage (%)
Age	20–40 years	43	49
	41–60 years	44	51
Gender	Male	40	46
	Female	47	54
Weight	≤80 kg	36	41
	>80 kg	51	59
Side of the shoulder	Left	38	44
	Right	49	56

The duration of complaints was categorized into ≤2 weeks for 33 (38%) patients and >2 weeks for 54 (62%) patients. The mean duration of complaints was three weeks (SD = ±1.27). Regarding the effectiveness of the treatment, 81 (93%) patients reported the treatment as effective, while 6 (7%) patients found it to be not effective (Table 2).

**Table 2:** Duration and effectiveness (n = 87).

Variable	Categories	Frequency	Percentage (%)
Duration of complaints	≤2 weeks	33	38
	>2 weeks	54	62
Effectiveness	Effective	81	93
	Not effective	6	7

In the age group of 20-40 years, 40 (93%) patients found the treatment effective, while 3 (7%) patients did not, with a p-value of 0.9767, indicating no significant difference in effectiveness between age groups. In the 41-60-year age group, 41 (93%) patients reported effectiveness, and 3 (7%) patients did not. Regarding gender, 38 (95%) male patients found the treatment effective, while 2 (5%) male patients did not, and 43 (91%) female patients reported effectiveness, with 4 (9%) female patients finding it ineffective. The p-value for gender was 0.5195, suggesting no significant difference in treatment effectiveness between males and females (Table 3).

**Table 3:** Stratification of effectiveness by demographic variables (n = 87).

Variable	Categories	Effective (%)	Not effective (%)	Total (%)	P-value
Age	20–40 years	40 (93%)	3 (7%)	43 (100%)	0.9767
	41–60 years	41 (93%)	3 (7%)	44 (100%)	
Gender	Male	38 (95%)	2 (5%)	40 (100%)	0.5195
	Female	43 (91%)	4 (9%)	47 (100%)	

Among participants weighing ≤80 kg, 35 (97%) found the treatment effective, while 1 (3%) did not, compared to 46 (90%) finding it effective and 5 (10%) not effective in those weighing >80 kg, with a p-value of 0.2027, indicating no significant difference based on weight. For the side of the shoulder, 35 (92%) left shoulder participants and 46 (94%) of right shoulder participants found the treatment effective, with a p-value of 0.7426, showing no significant difference between the two groups. Regarding the duration of complaints, 32 (97%) patients with complaints lasting ≤2 weeks found the treatment effective, while 1 (3%) did not, compared to 49 (91%) finding it effective and 5 (9%) not effective in those with complaints lasting >2 weeks, with a p-value of 0.2659, indicating no significant difference based on the duration of complaints (Table 4).

**Table 4:** Stratification of effectiveness by other variables (n = 87).

Variable	Categories	Effective (%)	Not effective (%)	Total (%)	P-value
Weight	≤80 kg	35 (97%)	1 (3%)	36 (100%)	0.2027
	>80 kg	46 (90%)	5 (10%)	51 (100%)	
Side of the shoulder	Left	35 (92%)	3 (8%)	38 (100%)	0.7426
	Right	46 (94%)	3 (6%)	49 (100%)	
Duration of complaints	≤2 weeks	32 (97%)	1 (3%)	33 (100%)	0.2659
	>2 weeks	49 (91%)	5 (9%)	54 (100%)	

The mean baseline VAS pain score was 7.2 ± 1.3, which reduced to 2.1 ± 1.1 at the three-week follow-up (p < 0.001), reflecting a mean pain reduction of 5.1 points. Functional improvement was reported by 81 out of 87 (93%) patients, based on subjective improvement in range of motion and daily activities.

No adverse events or complications, including infection, allergic reactions, or post-injection flare, were reported by any of the 87 patients during or after the procedure, or at the three-week follow-up visit.

## Discussion

The rotator cuff is a group of muscles made up of the subscapularis, infraspinatus, teres minor, and supraspinatus muscles, allowing a wide range of motion and stability to the glenohumeral joint [1]. Inflammation of the tendons of these muscles is called rotator cuff tendonitis [2]. It is often present along with shoulder impingement [11], and is one of the frequent complaints of patients presenting with shoulder pain, which may present acutely after an injury or due to repetitive overuse joint activity [10].

In our study population, the mean age was 46 ± 12.10 years, with a slightly higher proportion of females (54%) than males (46%). This demographic distribution is consistent with previous studies, such as Akbari et al. [5] and Murphy and Carr [6], which also reported a higher prevalence of rotator cuff disorders among middle-aged and older adults, particularly females. The increased incidence in females may be partially attributed to hormonal factors, occupational roles, and a greater likelihood of reporting musculoskeletal symptoms.

The predominance of right shoulder involvement (56%) observed in our study aligns with the findings of Alvarez et al. [7], where dominant-side involvement, usually the right, was more frequently noted, likely due to overuse in daily activities. The mean symptom duration in our cohort was approximately three weeks, which is shorter compared to studies involving chronic tendinopathy populations, such as Shin et al. [8], where symptom duration extended to several months. This shorter duration may have contributed to the higher short-term effectiveness observed in our findings.

Our patient population had a mean weight of 80 kg, with 59% weighing over 80 kg. While few studies have directly correlated body mass index (BMI) with injection outcomes, excess weight has been suggested as a potential risk factor for shoulder impingement and may influence treatment response. Future studies should consider analyzing BMI or obesity status as a stratifying variable.

Our study correlates with another study conducted by Murphy and Carr [6], in which subacromial steroid injection with lignocaine was effective in 90% of patients with rotator cuff tendonitis. Similar results were observed in the study conducted by Alvarez et al. [7], in which subacromial steroid injection with lignocaine was more effective at 93% compared to oral diclofenac potassium at 82% in terms of good functional outcomes among patients with rotator cuff tendonitis. In another study conducted by Shin et al. [8], in patients who were administered an injection, the mean (±SD) VAS for pain (P-VAS) score was 7.7 ± 1.2 at the time of the injection [12]. This significantly decreased to 2.3 ± 1.4 at the end of the first month after the injection, demonstrating a 70.2% reduction in pain (p < 0.01). At three months after the injection, the mean P-VAS score was 1.2 ± 1.8. Functional outcomes at final follow-up showed no significant differences between patients with and without an injection (American Shoulder and Elbow Surgeons score: 90.1 ± 14.6 with injection, 91.9 ± 8.2 without injection (p = 0.91); Constant-Murley Score: 89.1 ± 12.9 with injection, 84.5 ± 13.0 without injection (p = 0.17)). Patients with an injection showed no significant increase in the retear rate (6.8% with injection, 18.4% without injection; p = 0.06). According to the tear pattern, L-shaped rotator cuff tears (41.8%) showed a higher occurrence of severe postoperative persistent pain. Preoperative shoulder stiffness was revealed as a predisposing factor for persistent pain (odds ratio = 0.2; p = 0.04) [13].

Several limitations of the study should be acknowledged. First, the sample size was relatively small, which may affect the generalizability of the findings to a larger population. This study was conducted at a single tertiary care center in Peshawar, which may limit the generalizability of the findings to other regions or populations with differing demographics, healthcare practices, or comorbidity profiles. To improve external validity, future studies should include multicenter cohorts from diverse geographic and socioeconomic backgrounds. Second, the study did not include a control group, which limits the ability to directly compare the efficacy of the injection with other treatment methods. Additionally, the follow-up period was short, making it difficult to assess the long-term effectiveness and any potential side effects or recurrence of symptoms. While the VAS provided a quantifiable measure of pain reduction, we acknowledge the lack of validated composite scores, such as the Shoulder Pain and Disability Index or repeated Constant-Murley Scores, as a limitation. Incorporating such tools would enhance the objectivity and reproducibility of outcomes in future research. It is important to note that all injections were administered using a landmark-guided technique. While this approach is commonly used in clinical practice, ultrasound-guided injections may offer improved accuracy and outcomes. Future studies comparing both techniques would be valuable. Finally, the study did not control for potential confounding factors, such as variations in the severity of rotator cuff tendonitis or comorbidities that could influence the outcome.

## Conclusions

The effectiveness of subacromial methylprednisolone acetate injection with lignocaine was 93% in rotator cuff tendonitis. This high success rate indicates that this treatment regimen can significantly alleviate pain and improve function in patients suffering from this common shoulder condition. The combination of methylprednisolone acetate, a corticosteroid, and lignocaine, a local anesthetic, provides both anti-inflammatory and analgesic effects, leading to effective management of symptoms.
